# Supplementary material for: Sustained Maternal Smoking Triggers Endothelial-Mediated Oxidative Stress in the Umbilical Cord Vessels, Resulting in Vascular Dysfunction
Source: Antioxidants (Basel). 2021 Apr 9;10(4):583. doi: 10.3390/antiox10040583 (PMC8069726; doi:10.3390/antiox10040583)
Supplement: Supplementary file 1 [file antioxidants-10-00583-s001.pdf]

Supplementary figures

Sustained maternal smoking triggers endothelial-mediated oxidative stress in the umbilical cord vessels, resulting in vascular dysfunction

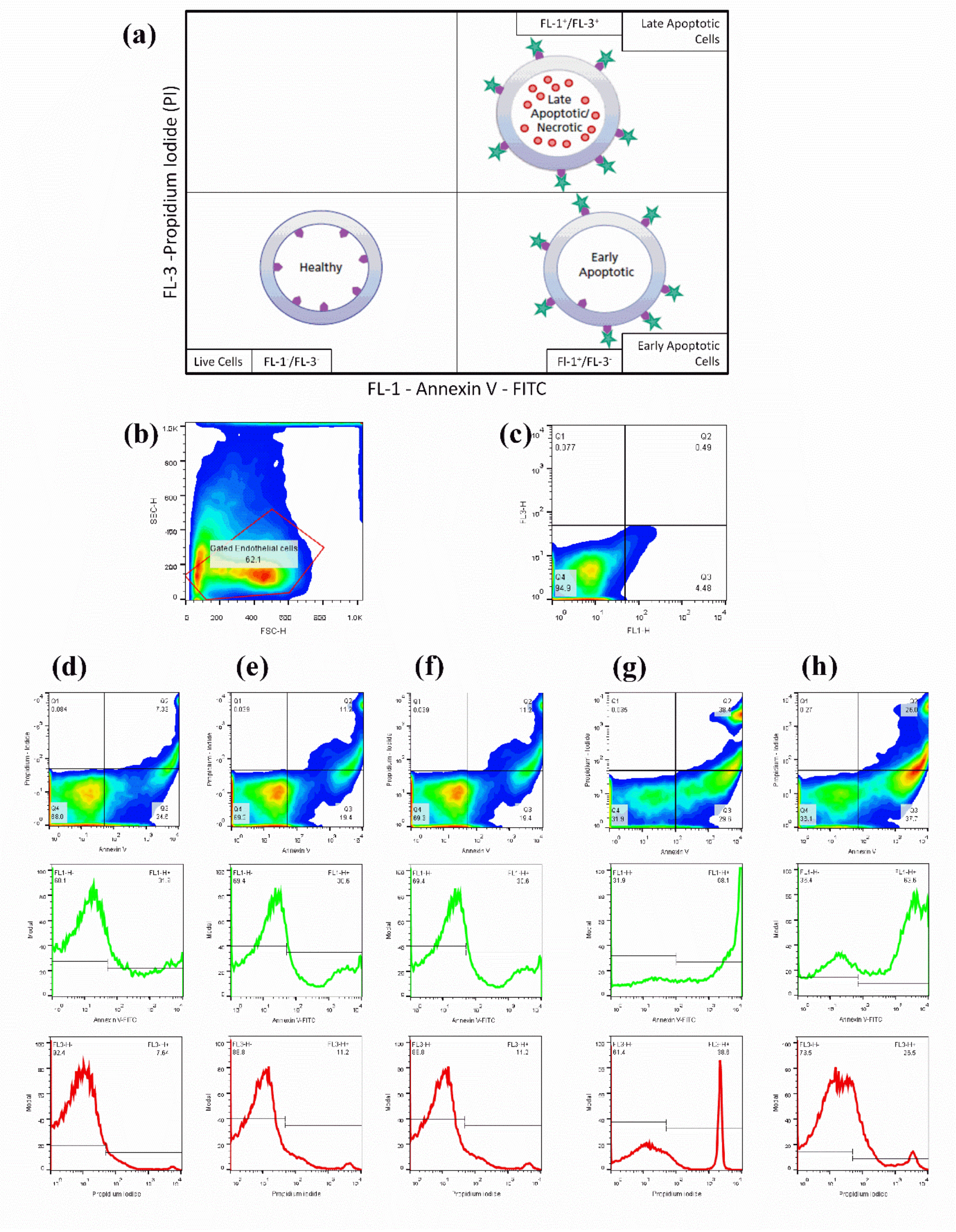

Figure S1. Detailed representation of viability assay with Annexin V-FITC and propidium iodide followed by FACS analysis. (**a**) Drawing of cell viability related properties and distribution among quadrants, based on Hingorani's and co-workers application note for BD Biosciences (Detection of Apoptosis Using the BD Annexin V FITC Assay on the BD FACSVerseTM System, 2011) [1]. (**b**) Raw cytogram of FACS result (FSC vs. SSC), where

the endothelial cell populations were gated out for each sample as an initial step [2]. (**c**) Raw cytogram of a blank sample. Quadrants were determined according to the FSC vs. SSC gated endothelial cells autofluorescence and used as a default setting for all the following evaluations. FL1-H is the Annexin V-FITC, FL3-H is the propidium iodide related channel of detection. (**d-f**) Results of gated endothelial cell populations derived from non-smoker samples. Upper images show the distribution of isolated endothelial cells among the four quadrants. Middle and bottom histograms present the Annexin V-FITC and propidium iodide intensities, plot against the cell number modality. (**g**, **h**) Results of gated endothelial cell populations originated from smoker samples. Upper images show the distribution of isolated endothelial cells among the four quadrants. Middle and bottom histograms present the Annexin V-FITC and propidium iodide intensities, plot against the cell number modality.

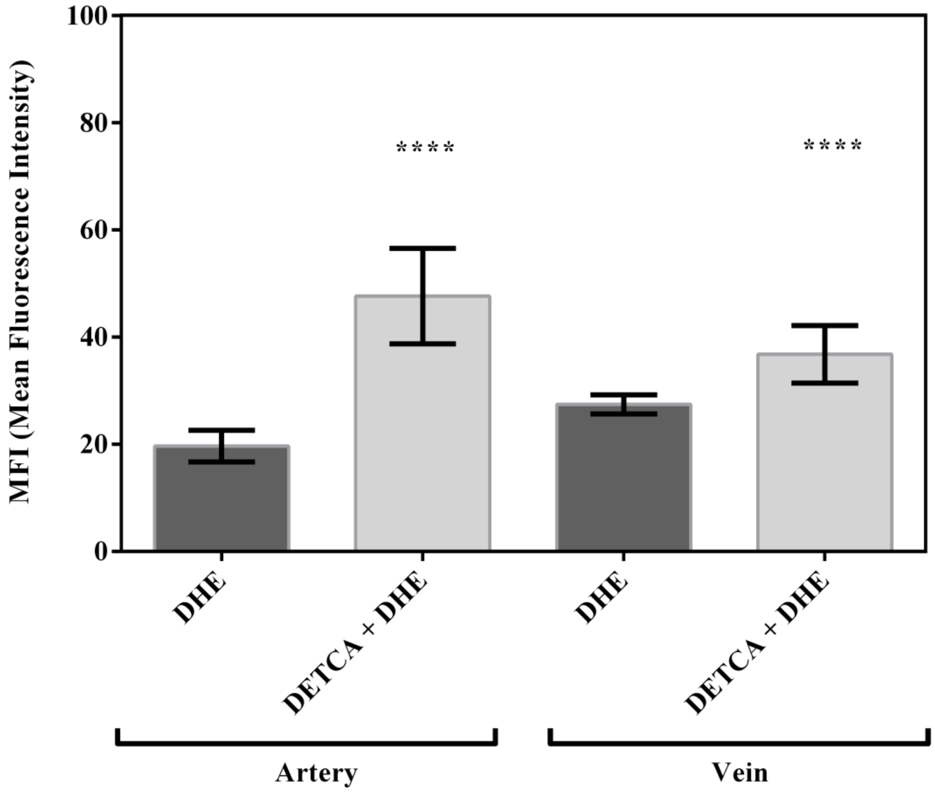

Figure S2. Semi-quantified data of nuclear intercalated oxDHE levels in UC arteries and veins +/- DETCA. Before DHE application, DETCA treatment was performed in order to inhibit the tissues' inner superoxide – dismutase activity, thus the intracellular $O_2^{\bullet -}$ levels [3]. MFI (mean fluorescent intensities) measured on consecutive sections at least in 5 independent fields of view as it described in the main text's material and methods section and the summarized data presented as mean ± SD. Sample numbers were the following DHE (n = 191 artery and 50 vein ROI from 2 individual samples); DETCA + DHE (n = 196 artery and 56 vein ROI from 2 individual samples). Statistics: unpaired t-test followed by Mann - Whitney test to compare ranks and ****$p \leq 0.0001$.
DHE: dihydroethidium, DETCA: diethyldithiocarbamate

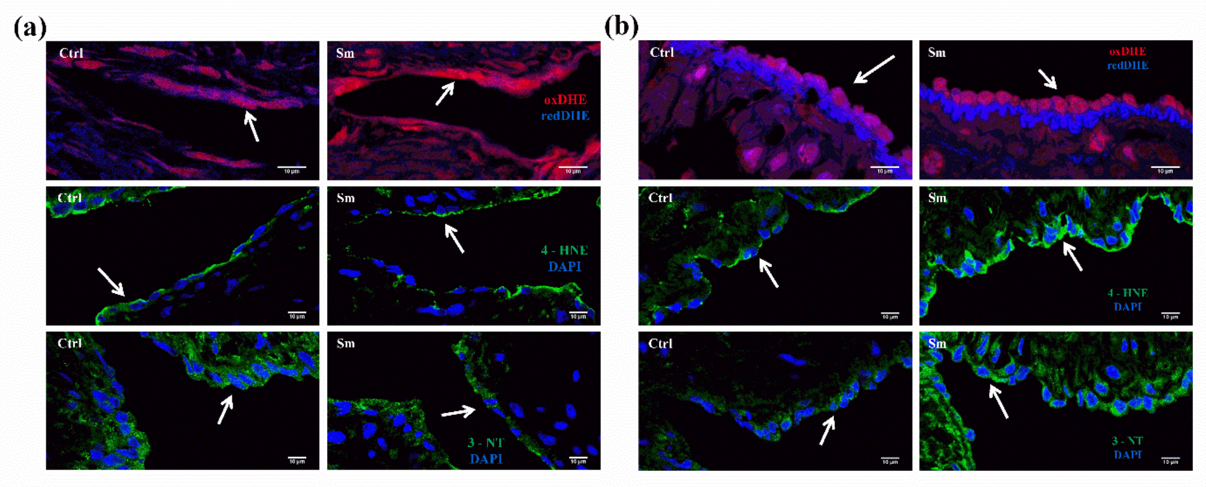

Figure S3. Oxidative stress markers in UC vessels, with control (Ctrl) and smoker (Sm) origins. Representative confocal images showing UC arteries (**a**) and veins (**b**) incubated with DHE (10 µM), 4-hydroxy-2-nonenal (4-HNE), and 3-nitrotyrosine (3-NT) antibodies, specific for macromolecular damages. Reduced DHE (redDHE) showing weak blue fluorescence in the cytoplasm, the oxidized DHE (oxDHE) showing strong red fluorescence, when intercalated into nuclei. Double immunolabeling were carried out with mouse monoclonal anti-4-HNE / 3-NT antibodies (1:100) followed by Alexa 488 (green) conjugated anti-mouse secondary antibody labelling (1:1000).

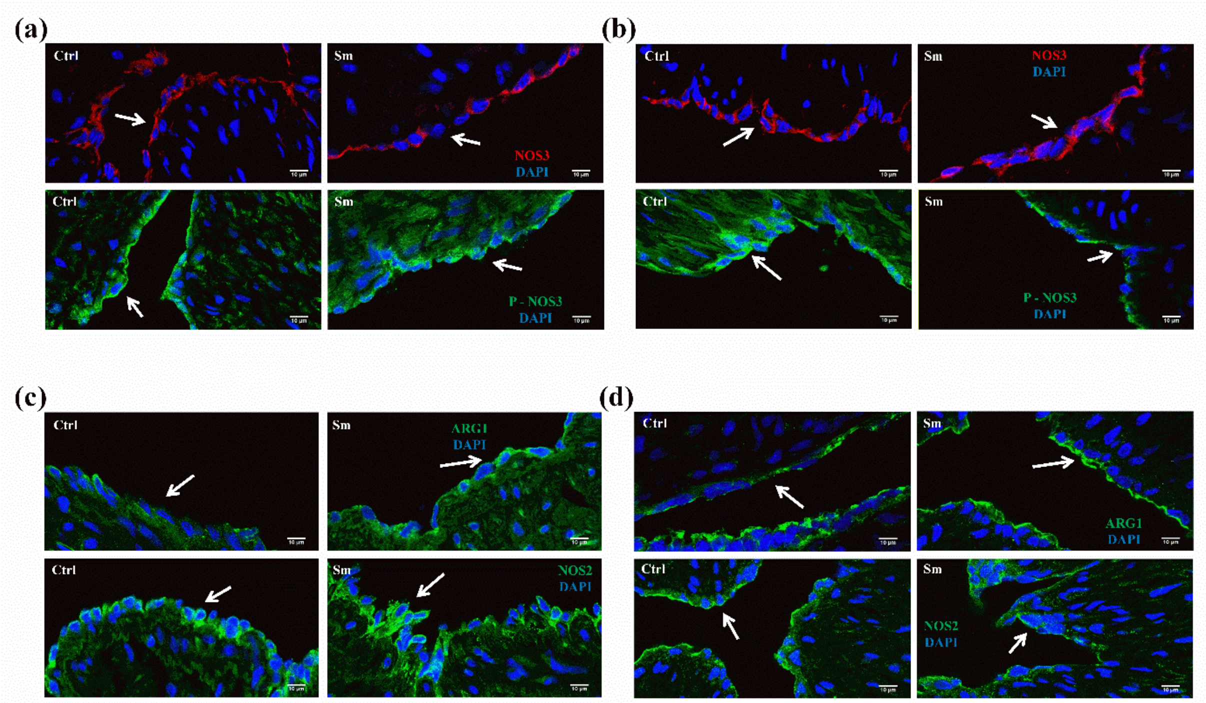

Figure S4. Following the NO synthesis determining factors in Ctrl and Sm UC vessels. Representative confocal images showing UC arteries (**a**) and veins (**b**) immunolabeled with mouse monoclonal anti-endothelial nitric oxide synthase (NOS3) or rabbit polyclonal P – NOS3 (1:100) followed by Alexa 647 (red) / 488 (green) conjugated anti – mouse / rabbit secondary antibody labelling (1:1000). The second panel showing UC arteries (**c**) and veins (**d**) double immunolabeled with mouse monoclonal anti Arginase-1/ inducible nitric oxide synthase (ARG1 / NOS2) (1:100) followed by Alexa 488 (green) conjugated anti – mouse secondary antibody labelling (1:1000).

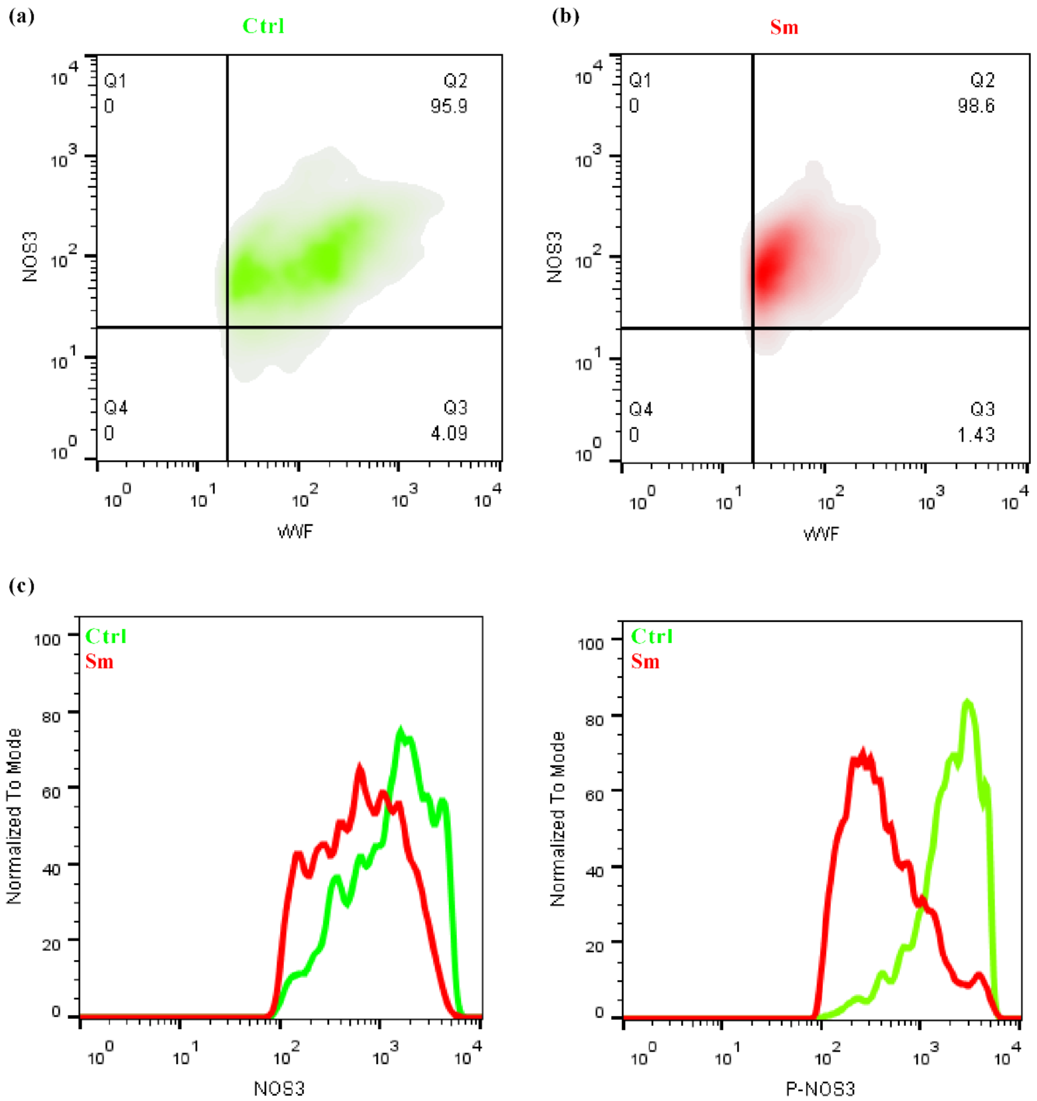

Figure S5. Quantitative FACS analysis dataset obtained from control and smoker derived isolated HUVEC population. (**a, b**) Purity validation of isolated cell suspension with double immunolabeling (anti-NOS3 and anti-von-Willebrand Factor). In the final results, supposed endothelial cell population were already gated out according to their (FSC vs. SSC), as it mentioned before [2]. Quadrants were determined according to the FSC vs. SSC gated cell's autofluorescence. Representative cytograms showing the fluorescent intensity distribution within the Ctrl and Sm derived isolates. The Q4 quadrant, regarded as the double positive endothelial population. The total cell numbers were the following: Ctrl: n=4960 cells from 3 samples; Sm: n= 4273 cells from 2 samples. (**c**) Histograms of anti-NOS3/anti-P-NOS3 immunolabeled HUVEC populations showing the fluorescent intensities against the cell number frequencies. The raw results gated out and the autofluorescence determined as mentioned above [2]. The total cell numbers were the following: Ctrl: n=4658 cells from 2 samples; Sm: n= 4305 cells from 2 samples

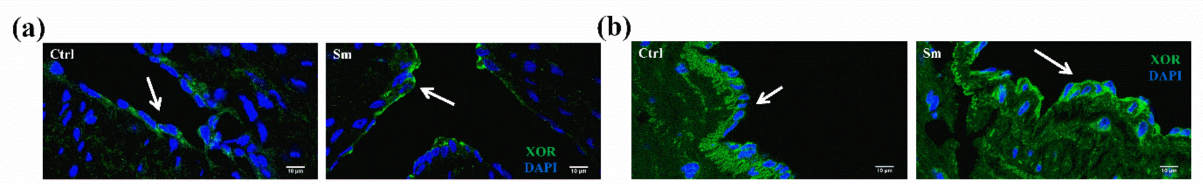

Figure S6. Following the NOS independent NO production in Ctrl and Sm UC vessels. Representative confocal images showing UC arteries (**a**) and veins (**b**) immunolabeled with mouse monoclonal anti – xanthine oxidoreductase (XOR) (1:100) followed by Alexa 488 (green) conjugated anti – mouse secondary antibody labelling (1:1000).

2. Supplementary references

1. Hingorani, R.; Deng, J.; Elia, J.; McIntyre, C.; Mittar, D. Detection of Apoptosis Using the BD Annexin V FITC Assay on the BD FACSVerse™ System. *BD Biosciences, San Jose* **2011**, 1–12.
2. Reiss, Y.; Engelhardt, B. FACS Analysis of Endothelial Cells. In *Methods in Endothelial Cell Biology*; Augustin, H.G., Ed.; Springer Lab Manuals; Springer: Berlin, Heidelberg, 2004; pp. 157–165 ISBN 978-3-642-18725-4.
3. Martin, W.; McAllister, K.H.; Paisley, K. NANC Neurotransmission in the Bovine Retractor Penis Muscle Is Blocked by Superoxide Anion Following Inhibition of Superoxide Dismutase with Diethyldithiocarbamate. *Neuropharmacology* **1994**, *33*, 1293–1301, doi:10.1016/0028-3908(94)90029-9.
